# Size dependent differences in litter consumption of isopods: preliminary results

**DOI:** 10.3897/zookeys.176.2470

**Published:** 2012-03-20

**Authors:** Ferenc Vilisics, Sándor Szekeres, Elisabeth Hornung

**Affiliations:** 1 Helsinki University, Faculty of Bio- and Environmental Sciences, Department of Environmental Sciences, P.O. Box 65. 00014, Helsinki, Finland; 2 Szent István University, Faculty of Veterinary Science, Department of Ecology, 1400 Budapest, P.O. Box 2, Hungary

**Keywords:** *Porcellio scaber*, *Porcellionides pruinosus*, microcosm experiment, litter consumption, leaf orientation

## Abstract

A series of experiments were applied to test how leaf orientation within microcosms affect consumption rates (Experiment 1), and to discover intra-specific differences in leaf litter consumption (Experiment 2) of the common isopod species *Porcellio scaber* and *Porcellionides pruinosus*. A standardised microcosm setup was developed for feeding experiments to maintain standard conditions. A constant amount of freshly fallen black poplar litter was provided to three distinct size class (small, medium, large) of woodlice. We measured litter consumption after a fortnight. We maintained appr. constant isopod biomass for all treatments, and equal densities within each size class. We hypothesized that different size classes differ in their litter consumption, therefore such differences should occur even within populations of the species. We also hypothesized a marked difference in consumption rates for different leaf orientation within microcosms. Our results showed size-specific consumption patterns for *Porcellio scaber*: small adults showed the highest consumption rates (i.e. litter mass loss / isopod biomass) in high density microcosms, while medium-sized adults of lower densities ate the most litter in containers. Leaf orientation posed no significant effect on litter consumption.

## Introduction

Ecosystem processes, such as decomposition, are greatly influenced by the taxonomic and functional diversity of assemblages (Tilman et al. 1997). At the same time, natural populations consist of phenotypically various individuals representing differences in traits such as sex, age and morphology. Such differences have significant ecological consequences, such as intra-specific niche divergence among sexes (Shine 1989) and cohorts (Polis 1984, Werner and Gilliam 1984). In spite of these facts, as Bolnick et al. (2003) have pointed out *„…the majority of articles on measuring species’ niche width make no mention of the fact that individuals of the same species may use different resources…”*, because ecologists consider individuals as interchangeable when creating models for species interactions (Bolnick et al. 2011).

Confirming the statements above, studies suggest significant intra-specific divergence in the diet of soil macro-arthropods through their isotopic signatures (Okuzaki et al. 2009, Pollierer et al. 2009). Recently, Semenyuk and Tiunov (2011) showed similar results on several millipede species, suggesting that millipedes may change diet with their age. As isotopic signature reflects the diet of an individual (De Niro and Epstein 1978), the above mentioned differences suggest measurable differences in food preferences within populations.

Here, we focus on intra-specific differences in food consumption of terrestrial isopod species. Woodlice are relatively long-lived invertebrates that belong to the same guild (soil- and litter-dwelling macro-decomposers) in every life stage: manca, juvenile, pre-adult, adult. Isopods are effective decomposers (e.g. Hassall et al. 1987), and have a potential role in habitat remediation, thus, ecological restoration (e.g. Loureiro et al. 2006, Snyder and Hendrix 2008).

Decomposition through isopods is a phenomenon rather frequently studied, particularly in laboratory experiments (e.g. Szlávecz 1993, Zimmer et al. 2005, Hättenschwiler and Bretscher 2001). However, the papers seldom take note on the used age or size of animals as factors which may affect decomposition.

In our experiments we focused on distinct size classes of isopods. Intra-population variation in body size (especially between medium-sized and large adults) may also be explained by distinct life history patterns. As such, cohort-splitting is a phenomenon by which some individuals of a certain cohort grow slowly while others grow faster. Young males and females may grow differently, e.g. invest more in growth than reproduction in the early periods (e.g. Grundy and Sutton 1989).

Distinct isopod size classes, whether they are related to true age differences or cohort-splitting, may also differ in leaf litter utilization. This assumption is based on the idea that different size classes probably show differences in their feeding preferences – due to their anatomical features, e.g. ontogenic development in mouth parts, mandible morphology (Jackson 1928, Schmalfuss 2008) – on a given leaf (soft tissues vs. hard veins). Wieser (1966) has shown a size dependent, two-phase function in assimilation at the isopod *Porcellio scaber* Latreille, 1804. The referred work, however, made distinction between very small larvae (up to 0.3 mm) and larger individuals.

We conducted preliminary observations to detect how leaf orientation affects decomposition rates. We assumed that structural differences of the abaxial and adaxial sides of leaves will show distinctions in decomposition patterns. Isopods aggregate under logs and leaf litter to hide, mate and feed (Sutton 1980). Such shelters often serve as food source as well. Leaves of most higher plants show dorsoventral differences in their adaxial and abaxial conformation representing characteristic structures and functions. For example, the epidermis and plant cuticle are thicker on the adaxial side while most stomata are located on the abaxial side. Given its relatively better accessibility, decomposition is more likely to affect the abaxial side first. This assumption, combined with the sheltering behaviour of isopods may lead to a greater mass loss in leaves placed with the abaxial side downwards.

Choosing species-poor ecosystems (e.g. urban areas) as model for our studies, we aimed to see how different size classes of frequent urban species, *Porcellio scaber* and *Porcellionides pruinosus* Brandt, 1833, contribute to litter mass loss in laboratory experiments. For *Porcellio scaber* we used two different sets of experiments applying different densities for each size classes.

In this paper we also present a detailed description of microcosm setup and experimental design. We added some new features such as a multi-layer plaster system to maintain constant humidity, and standard order of leaf orientation to avoid biases from selective feeding.

### Hypotheses

**Figure 1.**
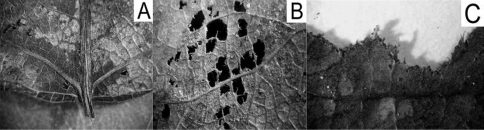
Patterns of litter degradation by *Porcellio scaber* size classes on poplar litter. A=small (0.2-0.5 mm in length), B=medium (0.7 – 10 mm), C=large (10 – 15 mm) isopods.

A pilot study showed differences in litter degradation patterns among size classes of *Porcellio scaber*: small bodied pre-adults primarily fed on leaf tissues amongst small veins, leaving vascular tissues and plant cuticle intact. Medium-sized adult individuals ate smaller veins and tissues, while large adults made no visible distinction in their choice (Fig. 1). Based on that observation, we assumed differences in litter consumption, too.

First, we hypothesize that leaf orientation affects consumption rates, explained by the general differences between abaxial and adaxial sides of tree leaves. We assume greater mass loss of leaves with abaxial side exposed, as abaxial side has thinner plant cuticle and protective layers (wax, hairs) than adaxial side.

Secondly, we hypothesized larger adults to consume more poplar litter due to their seemingly wider spectrum of food sources compared to the smaller ones.

## Methods

To test our two hypotheses, two separate microcosm experiments were set up. We established controlled conditions at the Institute for Biology at Szent István University, Budapest. To maintain constant humidity and temperature, we built a tent out of transparent plastic sheets and a wooden frame (5 m in length, 3 m in width, 2 m in height). The inner mean temperature was 19 ºC (SD±0.7), average relative air humidity was 68% (min. 61%, max 72%). We set a light regime of 12 / 12 hours of night and day.

### Microcosms

**Figure 2.**
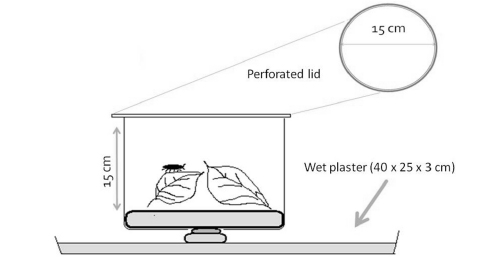
Schematic figure of a microcosm showing dimensions of the transparent plastic container and the plaster layer underneath.

For the experiments we used a set of microcosms assembled in the same way (Fig. 2). We used transparent poly-ethylene containers of 15 cm in height and 15 cm in diameter, with removable lids perforated for air exchange. To maintain constant humidity, we applied layers of plaster of Paris.

Solid plaster has already proven to be useful in laboratory experiments (e.g. Hassall et al. 1987, van Vliet et al. 1993): its water holding capacity maintains a humid environment in the container without making it too moist. To avoid water film on the surface (and e.g. fungal infection) we watered the containers from the bottom through additional layers of plaster of Paris. This system provided suitable humidity without getting too wet.

The humidity system consisted of three layers of plaster of Paris: a 3 cm thick bottom layer, a 0.5 cm bridging layer and a 1 cm thick contact layer. Plastic trays (bottom layer), shallow cups (bridging layer) and the containers for microcosm (contact layer) served as mold casting (see Fig. 2 for details).

The bottom layer was a slab of 40 cm x 30 cm plaster molded in a plastic tray watered directly. A hole was carved from one corner to monitor water level. As this layer should be always loaded with water, refill was necessary every second days (ca. 1.5 L / tray). As water does not always distribute evenly within plaster, it is important to maintain even surfaces.

The bridging layer, a thin disc of plaster, was placed between the watery bottom layer and the contact layer within the container. This layer was attached to the bottom of each container and transferred water to the contact layer through a hole cut on the bottom of each container. The bridging layer was glued to the contact layer by plaster. The contact layers, molded from microcosm containers, therefore precisely fit, were the solid grounds for isopods where litter consumption took place.

As preliminary studies showed a tendency for cannibalism (approx. 5% mortality), we decided to apply inedible shelters. For this purpose we used non-translucent rubber tubes (2 cm long, 0.5 cm diameter), 3 pieces in each container. Microcosms were placed randomly on trays of bottom layers.

### Leaf litter

For both experiments we used leaf litter of *Populus nigra* L. collected one week after fall and stored in the lab under dry conditions.The chosen tree species is common in Hungarian urban green spaces and floodplain forests. Leaf litter was collected from a major park (Városliget) in Budapest. We used leaves of similar stage of decay: surface and edge were unbroken, the colour was brown. After washing off the dust, we removed the petioles from the leaves. Litter was oven dried at 60°C for 20 hours. Litter processing varied between experiments as described in sub-chapters below.

### Test species

We chose the common rough woodlouse *Porcellio scaber* and *Porcellionides pruinosus* as test species for our experiments. *Porcellio scaber* is a frequently used organism of laboratory research (e.g. Farkas et al. 1996, Ihnen and Zimmer 2008). Both species are frequent in urban environments and occur all over the world (Schmalfuss 2003).

*Porcellio scaber* individuals were collected in an enclosed garden in Hajdúböszörmény for Experiment 1, and in central Budapest for Experiment 2. Individuals of *Porcellionides pruinosus* were collected from a compost heap in the park of the Faculty of Veterinary Sciences, Szent István University, Budapest.

As isopods gut content may differ, we attempted to let the animals empty their guts without refilling it. All animals were starved for two days and weighed before and after the experiment with a digital analytical scale.

It is known that a single population may produce great annual, or even seasonal variations (e.g. Stearns 1992) in its metabolism. As all of our experiments were conducted in the similar period of the year (winter and early spring) period, under controlled (temperature, humidity, photoperiod) conditions, we consider our data comparable.

### Data analyses

To test our hypotheses on leaf litter consumption we used Mood’s median test as implemented in R (R Development Core Team 2012). The null-hypothesis of this robust, non-parametric test assumes that the medians of two data sets are not different. To calculate leaf litter consumption rates we divided litter mass loss (mg) by the average of the initial and final weights of isopods per microcosm. With this method we got the litter mass loss milligram per isopod milligram. Data of microcosms with a mortality higher than 20% were omitted.

### Experiment 1: Effects of leaf orientation on litter consumption

To reveal the effects of different leaf orientation (adaxial side up or down) in litter consumption experiments, we placed standard discs (ca 2 cm in diameter) cut from *Populus nigra* leaves in microcosms (c.f. Loureiro et al. 2006). With 13 replicates we used leaf discs with their abaxial side up, while in another 13 containers abaxial side down. Dry weights were measured after five days and compared to initial oven-dried dry weights for each container. As mortality in each container occurred in less than 20% of individuals, we included data from all containers to the analyses.

To the experiment either five *Porcellio scaber* (size >1 cm, 238 mg ± 5 mg) or five *Porcellionides pruinosus* (cca. 0.5 cm, 71 mg ± 2 mg) adult individuals were used per container.

### Experiment 2: Intra-specific, size dependent litter consumption rates

To each microcosm we added 1500 mg (± 1 mg) of poplar leaves, three pieces per container. As we supposed that litter orientation biases consumption rates, we arranged leaves in a standard order: 1^st^ leaf abaxial side down, 2^nd^ leaf adaxial side down, 3^rd^ leaf abaxial side down, etc.

Litter dry mass was measured by a digital analytical scale at the start, and after 14 days of experimental period (with 12 hours light-dark regime). Faecal pellets and dirt were brushed off prior to final weighing after oven-drying (60 °C) for 20h.

Out of the collected individuals we selected three distinct size classes (small: 3-5 mm, medium: 7-11 mm, large: >15 mm).

Within Experiment 2, we used two experimental settings by using different isopod densities within microcosms. We attempted to use similar numbers of isopod individuals in similar weights at each size class per each replicate (microcosm). Mean numbers, biomasses and the number of microcosms used in our experiments are shown in Table 1.

**Table 1.** Mean (±SD) number of isopod individuals, their cumulated biomasses and the number of microcosms used in the experiments.

Size category		Experiment 2/a				Experiment 2/b	
		*Porcellionides pruinosus*		*Porcellio scaber*		*Porcellio scaber*	
Small	No. of ind.	20	(SD±0)	12	(SD±0)	51	(SD±5.9)
	Biomass (mg)	82.5	(SD±1.87)	224.3	(SD±14.76)	301.1	(SD±2.76)
Medium	No. of ind.	10	(SD±0)	6	(SD±0)	8.9	(SD±0.76)
	Biomass (mg)	85	(SD±3.35)	166	(SD±2.74)	303.7	(SD±8.67)
Large	No. of ind.	5.2	(SD±0.24)	3	(SD±0)	4.7	(SD±0.23)
	Biomass (mg)	83.5	(SD±2.26)	232.8	(SD±11.29)	299.9	(SD±2.89)
Number of microcosms analysed		8		9		10	

Legend: No. of ind. = Number of individuals within microcosms; Biomass = isopod biomass (mean±SD) within microcosms

At Experiment 2/a we attempted to keep isopod biomass constant at around 200 mg in each microcosm, regardless to size classes. Whereas in Experiment 2/b we used only *Porcellio scaber* individuals in densities higher than in Exp. 2/a. In this case, isopod biomass was kept at around 300 mg in each microcosm for all size classes.

Data of containers with a mortality rate higher than 20% were omitted from analyses. In order to keep similar sample sizes, we had to omit data from other size classes as well (even if their mortality rate was lower than 20%). In such cases we used a random number generator by which we selected data for deletion. This practice has also resulted in differences in numbers of replicates among Experiments, as seen on Table 1.

## Results

### Experiment 1: Effects of leaf orientation on litter consumption

**Figure 3.**
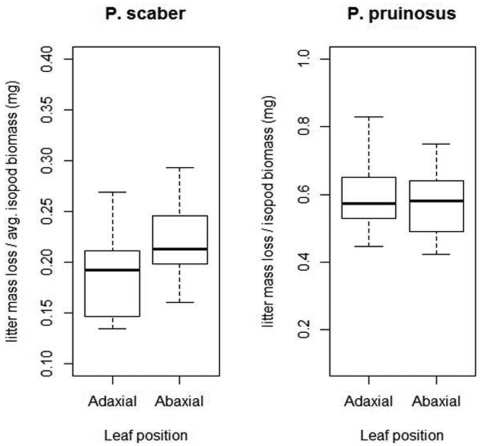
Effects of leaf litter positioning (abaxial up vs. abaxial down) on litter consumption of two isopod species. Note the different scaling of y axes. Legend: P. pruinosus: *Porcellionides pruinosus*; P. scaber: *Porcellio scaber*; Adaxial = adaxial side up; Abaxial = abaxial side up; Thick line = median; box = lower and upper quartiles; whisker = min. and max values.

With *Porcellio scaber* we found visible, albeit not significant (Mood’s median test, p=0.11), differences in litter mass loss (Fig. 3). Similarly, Mood´s median test revealed no significant effect of leaf orientation on the litter consumption rates of *Porcellionides pruinosus* (p=1).

### Experiment 2/a and 2/b: Intra-specific differences in litter consumption

**Figure 4.**
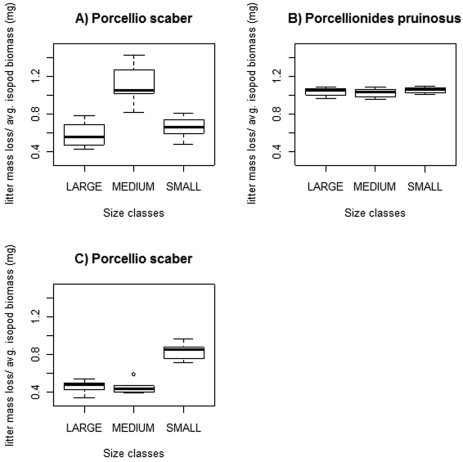
Freshly fallen poplar litter consumption of three size classes of *Porcellio scaber* and *Porcellionides pruinosus*. Legend: Thick line = median; box = lower and upper quartiles; whisker= min. and max values; open circles: outliers.

Litter mass loss differed among the three size classes of *Porcellio scaber* at both experimental sets (2/a,b), while no significant differences were found among classes of *Porcellionides pruinosus*. Figure 4 shows the main results: A and B represent Experiment 2/a, while C represents Experiment 2/b.

In Experiment 2/a (lower *Porcellio scaber* densities) we measured statistically significant differences between „medium” and both „large” and „small” classes (Fig. 4/A). The latter two categories showed no statistically significant difference in their litter consumption, (p=0.131). *Porcellionides pruinosus* size classes showed no marked difference in their litter consumption (p=1).

In Experiment 2/b (the setting with higher *Porcellio scaber* densities) we measured significant differences between the „small” and both „medium” and „large” classes (Mood’s median test, p<0.001), while the two larger categories showed no significant difference (p=1).

All *Porcellionides pruinosus* size classes consumed, in general, ca. 1 mg_leaf_ / 1 mg_isopod_, while this rate was less in most *Porcellio scaber* size classes in both experimental settings (Fig. 4).

## Discussion

The study proved that (with the current setup) leaf orientation do not have significant effect on leaf litter consumptions. Isopod size classes, to certain degrees however, can bias leaf litter consumption rates in microcosm experiments.

It is evident that at this stage of our research we are unable to explain the reasons of the patterns we got. Therefore we devote most of this chapter to speculations.

Leaf orientation, which had - to the best of our knowledge - never been studied before, has proven to pose no effect in biasing consumption rates. Our approach applies for microcosm experiments using small number of leaves (cc. 1–3) where „random” arrangement is not possible. We assume that greater differences would appear in consumption rates between the two sides as soon as isopods could reach the bottom of leaf litter. Woodlice normally hide under dead plant matter, using it as shelter and food source at the same time (Stachursky 1968). The top of a leaf may be exposed to predators and other danger, so feeding on the surface may not be natural for isopods.

Leaves exposed to sun are large and thin while small and thick leaves develop in the shade (e.g. Jackson 1967). Sun leaves have a more developed spongy and palisade mesophyll regions, and higher photosynthetic rates in comparison to the shadow leaves on the same tree (e.g. Nobel 1976). Several studies prove that solar radiation activates flavonoid biosynthesis resulting sun leaves to contain higher amounts of phenolics (e.g. Jaakola et al. 2004). At the same time, litter quality differs between urban and rural habitats (McDonnell et al. 1997). These facts suggest that selecting the right leaves may be of great importance, as well.

Opposite to our hypothesis, large *Porcellio scaber* individuals ate relatively less than smaller adults. In fact, large adults consumed very little of the leaf litter. For this reason we suspect that the primary food source of this species may be something more palatable than freshly fallen (or near-freshly fallen) litter. The relatively large consumption rates for smaller (small- and medium-sized) adults is probably explained by their higher metabolism induced by intense growth. This agrees with the findings of Reichle (1968) who showed an inverse correlation between metabolic rates and live body weights for different arthropod species (i.e. smaller arthropod species had higher metabolic rates). Pennington and Meehan (2007) have, however, shown that metabolic rates of centipedes had a positive correlation with body mass. Still, as intra-population variations of the metabolic rates of other Porcellionid species (*Porcellio laevis* Latreille, 1804) is also known (Lardies and Bozinovic 2008), we should regard that phenomenon as of high importance in understanding patterns of intra-population litter consumption by woodlice. Still, the reason why *Porcellionides pruinosus* size classes displayed nearly equal consumption patterns remains unanswered.

Our results with *Porcellio scaber* suggest that mainly the small individuals contribute in the comminution process of leaf litter. This function may be especially valuable in areas with low soil activity and species poor decomposer fauna, such as urban areas (e.g. McDonnell et al. 1997). Size dependent litter mass loss and functional differentiation within populations may be estimated by combining seasonal activity and demography data of natural populations with in-vitro litter mass loss rates.

Besides the effects of size classes, we have also shown results more likely related to densities (individuals per container) than isopod sizes (Rushton and Hassall 1987). Based on our results, we suppose that density poses a substantial effect on litter mass loss in microcosm experiments. On the analogy to the Allee-effect (e.g. Stephens et al. 1999), we think that litter mass loss increases with an increasing density until it reaches a point where intra-population competition stabilizes or even decreases consumption rates.

## Conclusions

With these results, we would like to show some details that can, to some degree, bias results in laboratory experiments. In order to provide more reliable results in microcosm experiments we suggest standardizing the size and density of isopods.
